# Comparison of fludarabine–melphalan and fludarabine–treosulfan as conditioning prior to allogeneic hematopoietic cell transplantation—a registry study on behalf of the EBMT Acute Leukemia Working Party

**DOI:** 10.1038/s41409-022-01646-1

**Published:** 2022-05-14

**Authors:** Jesus Duque-Afonso, Jürgen Finke, Myriam Labopin, Charles Craddock, Rachel Protheroe, Panagiotis Kottaridis, Eleni Tholouli, Jenny L. Byrne, Kim Orchard, Urpu Salmenniemi, Inken Hilgendorf, Hannah Hunter, Emma Nicholson, Adrian Bloor, John A. Snowden, Mareike Verbeek, Andrew Clark, Bipin N. Savani, Alexandros Spyridonidis, Arnon Nagler, Mohamad Mohty

**Affiliations:** 1 Department of Hematology/Oncology, Faculty of Medicine, University of Freiburg Medical Center, Freiburg, Germany; 2 EBMT Paris Study Office, Hopital Saint Antoine, Paris, France; 3 Birmingham Centre for Cellular Therapy and Transplantation, Queen Elizabeth Hospital Birmingham, Edgbaston, Birmingham UK; 4 University Hospitals Bristol and Weston NHS Foundation Trust, Bristol, UK; 5 Department of Haematology, University College London Hospital, London, UK; 6 Clinica Haematology Department, Manchester Royal Infirmary, Manchester, UK; 7 Nottingham University, Hucknall Road, Nottingham, UK; 8 Wessex Blood and Marrow Transplant and Cellular Therapy Program, University Hospital Southampton NHS Foundation Trust, Southampton, UK; 9 Stem Cell Transplantation Unit, HUCH Comprehensive Cancer Center, Helsinki, Finland; 10 Universitaetsklinikum Jena, Klinik für Innere Medizin II, (Abt. Hämatologie und Onkologie), Am Klinikum 1, Jena, Germany; 11 University Hospitals Plymouth NHS Trust, Derriford Hospital, Plymouth, UK; 12 Department of Haematology, Royal Marsden Hospital, London, UK; 13 The Christie NHS Foundation Trust, Stem Cell Transplantation Unit, University of Manchester, Manchester, UK; 14 Sheffield Teaching Hospitals NHS Trust, Royal Hallamshire Hospital, Sheffield, UK; 15 Klinikum Rechts der Isar, III Med Klinik der TU, Munich, Germany; 16 Bone Marrow Transplant Unit, Beatson, West of Scotland Cancer Centre, Gartnaval General Hospital, Glasgow, UK; 17 Division of Hematology and Oncology, Vanderbilt University Medical Center, Nashville, TN USA; 18 University Hospital of Patras, Patras, Greece; 19 Hematology Division, Chaim Sheba Medical Center, Tel Hashomer, Israel; 20 Sorbonne University, Saint-Antoine Hospital, AP-HP, INSERM UMRs 938, Paris, France

**Keywords:** Translational research, Stem-cell research

## Abstract

In recent years considerable variations in conditioning protocols for allogeneic hematopoietic cell transplantation (allo-HCT) protocols have been introduced for higher efficacy, lower toxicity, and better outcomes. To overcome the limitations of the classical definition of reduced intensity and myeloablative conditioning, a transplantation conditioning intensity (TCI) score had been developed. In this study, we compared outcome after two frequently used single alkylator-based conditioning protocols from the intermediate TCI score category, fludarabine/melphalan 140 mg/m^2^ (FluMel) and fludarabine/treosulfan 42 g/m^2^ (FluTreo) for patients with acute myeloid leukemia (AML) in complete remission (CR). This retrospective analysis from the registry of the Acute Leukemia Working Party (ALWP) of the European Society of Bone Marrow Transplantation (EBMT) database included 1427 adult patients (median age 58.2 years) receiving either Flu/Mel (*n* = 1005) or Flu/Treo (*n* = 422). Both groups showed similar 3-year overall survival (OS) (54% vs 51.2%, *p* value 0.49) for patients conditioned with FluMel and FluTreo, respectively. However, patients treated with FluMel showed a reduced 3-year relapse incidence (32.4% vs. 40.4%, *p* value < 0.001) and slightly increased non-relapse mortality (NRM) (25.7% vs. 20.2%, *p* value = 0.06) compared to patients treated with FluTreo. Our data may serve as a basis for further studies examining the role of additional agents/ intensifications in conditioning prior to allo-HCT.

## Introduction

Allogeneic hematopoietic cell transplantation (allo-HCT) protocols are in continuous evolution and are constantly being evaluated for higher efficacy, lower toxicity, and better outcomes [1]. The optimal conditioning regimen for patients with several co-morbidities and/or organ toxicity prior to allo-HCT is a focus of intense research [2–4]. Recent data from the Acute Leukemia Working Party (ALWP) of the European Society of Bone Marrow Transplantation (EBMT), showed that conditioning intensity should be considered as a continuum rather than be defined as “classical” reduced intensity conditioning (RIC) or myeloablative conditioning (MAC) and a transplantation conditioning intensity (TCI) score had been developed [5]. In this study, we aimed to assess the outcome after conditioning with a specific single alkylating agent with fludarabine containing protocols from the intermediate intensity (TCI score 2.5–3.5) category.

Conditioning with fludarabine/melphalan (FluMel) has been suggested to have significant anti-leukemia activity while showing moderate toxicity [6, 7]. Patients receiving FluMel (total melphalan dose ranging from 130 to 150 mg/m^2^) showed lower relapse incidence and slightly higher non-relapse mortality (NRM) compared to patients treated with lower-intensity fludarabine/busulfan (FluBu2, total busulfan dose orally 7.1–8.9 mg/kg and intravenously 6.0–6.9 mg/kg for 2 days), resulting in similar leukemia-free survival (LFS) and overall survival (OS) [8]. Treosulfan-based protocols (total dose ranging from 30 to 42 g/m^2^) have been shown to be safe and effective and are good alternatives to busulfan or total body irradiation (TBI)-based regimens in the conditioning for acute myeloid leukemia (AML) treatment [9]. In recent years, conditioning with fludarabine/treosulfan (FluTreo) has been increasingly used in conditioning protocols for adult patients. This protocol had been developed for pediatric patients and its dosage has been optimized for adults with a treosulfan total dose of 42 g/m^2^, showing the lowest relapse rate with similar toxicity [10, 11]. In a single center retrospective analysis with a limited number of patients (*n* = 138) FluMel (total dose 140 mg/m^2^) and FluTreo (total dose 36–42 g/m^2^) conditioning protocols were compared. Patients conditioned with FluMel suffered more frequently with grade III–IV oral mucositis and diarrhea but had similar outcomes to patients conditioned with FluTreo [12]. Nevertheless, the most frequently used conditioning protocols within the intermediate TCI score category such as FluMel and FluTreo have not been compared in clinical and/or in large registry studies, or in a homogenous patient population.

We therefore used the registry of the EBMT to retrospectively analyze data from patients treated with two of the most used conditioning protocols with an intermediate TCI score, based on the single alkylating agent combinations of FluMel and FluTreo. Outcome variables included OS, LFS, relapse incidence, NRM, graft-versus-host disease (GvHD), and GvHD-free, relapse-free survival (GRFS). Characteristics of patients, donor type, cause of death, complications including incidence of GvHD, as well as immunosuppression and GvHD prophylaxis were also analyzed.

## Methods

### Study design

In this retrospective multicenter analysis, data were provided by the ALWP of the EBMT in which >600 transplant centers report annually all consecutive allo-HCTs after patient authorization via informed consent, and approval of the study from the ALWP EBMT general assembly. We focused on (1) adult (aged > 18 years) patients who received conditioning with FluMel (fludarabine 150 mg/m^2^, melphalan 140 mg/m^2^) or with FluTreo (fludarabine 150 mg/m^2^, treosulfan 42 g/m^2^), (2) first allo-HCT from a matched sibling donor (MSD) or matched unrelated donor (MUD 10/10 and 9/10—including HLA-A, -B, -C, or -DRB1 and DQB1 mismatches -) for patients with AML including secondary AML in complete remission (CR), (3) transplantation date between January 1st, 2008 and December 31st, 2018, (4) with an unmanipulated peripheral blood stem cell graft (no in vitro T-cell depletion (TCD)). Patients undergoing haplo-identical or cord blood allo-HCT were excluded. CR was defined as less than 5% blasts in bone marrow at the time of allo-HCT. GvHD prophylaxis was conducted according to local institutional guidelines. Doses and sources were not always documented in the ALWP registry. Post-transplant events such as hematological relapse, GvHD among others were defined based on standard clinical and laboratory criteria. All patients gave signed informed consent for data submission and scientific analysis within the registry. The study was approved by the general assembly of the ALWP of the EBMT.

### Statistical analysis

Outcome variables were defined following internal consensus guidelines [13]. Patient-, disease-, and treatment-related characteristics were compared using the chi-square test for categorical data or the Mann-Whitney test for continuous data. Baseline characteristics were summarized using median, interquartile range (IQR), and range, for continuous data, and frequency and percentage for categorical data. OS was defined as the time from allo-HCT until death from any cause. LFS was defined as the time from allo-HCT to death from any cause, or relapse, whichever occurred first. Relapse was defined as detection of disease via cytological and histological assessment after allo-HCT; death without prior relapse was considered as a competing risk for relapse and was denoted as NRM. For cumulative incidence of acute GvHD (aGvHD) and chronic GvHD (cGvHD), death without aGvHD/cGvHD and relapse were considered as competing events. GRFS was defined as being alive with neither grade III–IV aGVHD nor severe cGVHD, relapse, or death from any cause during the first year post-HCT. Patients with no event were censored at the date of last follow-up. To allow for the difference in follow-up period between the two conditioning regimen groups, outcome was censored at 3 years post transplantation for all comparisons. Planned subgroup analyses were performed for patients aged <55 years and ≥55 years.

Univariate analyses were performed using Gray’s test for cumulative incidence functions and the log-rank test for OS, GRFS, and LFS. The Cox proportional-hazards model was used for multivariate regression analysis, and included variables with unbalanced distribution between the two groups, those associated with an endpoint in univariate analysis, or factors known to predict outcomes. To allow for center differences, a random effect or frailty was introduced for each center into the models. Results were expressed as the hazard ratio (HR) with the 95% confidence interval (95% CI).

All tests were two sided. The Type I error was fixed at 0.05 for factors associated with time-to-event outcomes. Statistics were performed with SPSS 25.0 (SPSS Inc., Chicago, IL, USA) and R 4.0.3R Core Team (2020). R: A language and environment for statistical computing. R Foundation for Statistical Computing, Vienna, Austria. https://www.R-project.org/).

## Results

### Patient and transplant characteristics

The patient and transplant characteristics of the 1427 AML patients are shown in Table 1. Prior to allo-HCT, 1005 patients received a conditioning with FluMel, and 422 patients with FluTreo. The median patient age was 58.2 years (IQR 51.5–63.7) and the median follow-up was 48.1 months (95% CI 43.5–50.8 months) for the entire cohort and did not significantly differ between the two groups. Seven hundred and sixty-six (53.7%) patients were male.

**Table 1:** Patient and transplant characteristics.

Variable	Entire cohort	FluMel	FluTreo	*p* value
*N* (%)	1427 (100)	1005 (70.4%)	422 (29.6%)	
Year allo-HCT median (min-max)	2014 (2009–2018)	2014 (2009–2018)	2015 (2009–2018)	0.001
Median Follow-up	48.05	50.85	40	0.04
(months) [95%CI]	[43.5–50.8]	[47.6–56.2]	[36.4–46.8]	
Patient age (years)				0.59
median (min–max)	58.2 (18.2–76.2)	58 (19.8–76.2)	58.6 (18.2–75.7)	
[IQR]	[51.5–63.7]	[51.5–63.7]	[51.5–63.4]	
Age group				0.35
age <55years	527 (36.9%)	379 (37.7%)	148 (35.1%)	
age ≥55 years	900 (63.1%)	626 (62.3%)	274 (64.9%)	
KPS score				0.4
- <90	256 (19%)	184 (19.6%)	72 (17.6%)	
- ≥90	1091 (81%)	755 (80.4%)	336 (82.4%)	
- missing	80	66	14	
Patient sex				0.022
- male	766 (53.7%)	559 (55.7%)	207 (49.1%)	
- female	660 (46.3%)	445 (44.3%)	215 (50.9%)	
- missing	1	1	0	
Donor sex				0.71
- male	941 (66.4%)	664 (66.7%)	277 (65.6%)	
- female	477 (33.6%)	332 (33.3%)	145 (34.4%)	
- missing	9	9	0	
Female to male combination				0.021
- No	1184 (83.2%)	818 (81.7%)	366 (86.7%)	
- Yes	239 (16.8%)	183 (18.3 %)	56 (13.3%)	
- missing	4	4	0	
AML diagnosis				<0.0001
- de novo	1182 (82.8%)	861 (85.7%)	321 (76.1%)	
- secondary AML	245 (17.2%)	144 (14.3%)	101 (23.9%)	
Cytogenetics				0.019
- favorable	58 (4.1%)	46 (4.6%)	12 (2.8%)	
- intermediate	653 (45.8%)	467 (46.5%)	186 (44.1%)	
- adverse	166 (11.6%)	101 (10%)	65 (15.4%)	
- NA/failed	550 (38.5%)	391 (38.9%)	159 (37.7%)	
Status at allo-HCT				0.009
- CR1	1103 (77.3%)	758 (75.4%)	345 (81.8%)	
- CR2+	324 (22.7%)	247 (24.6%)	77 (18.2%)	
Patient CMV				<0.0001
- neg	475 (33.5%)	386 (38.7%)	89 (21.2%)	
- pos	942 (66.5%)	611 (61.3%)	331 (78.8%)	
- missing	10	8	2	
Donor CMV				0.041
- neg	690 (49%)	503 (50.8%)	187 (44.8%)	
- pos	717 (51%)	487 (49.2%)	230 (55.2%)	
- missing	20	15	5	
Donor type				<0.0001
- MSD	596 (41.8%)	470 (46.8%)	126 (29.9%)	
- MUD 10/10	651 (45.6%)	428 (42.6%)	223 (52.8%)	
- MUD 9/10	180 (12.6%)	107 (10.6%)	73 (17.3%)	
GvHD prophylaxis				
**-** CsA	780 (54.9%)	772 (77.4%)	8 (1.9%)	
- CsA + MTX	395 (27.8%)	103 (10.3%)	292 (69.2%)	
- CsA + MMF	177 (12.5%)	92 (9.2%)	85 (20.1%)	
- Other	68 (4.7%)	31 (3.1%)	37 (8.7%)	
- missing	7	7	0	
In vivo TCD				
- no in vivo TCD	224 (15.7%)	95 (9.5%)	129 (30.6%)	<0.0001
- in vivo TCD	1201 (84.3%)	908 (90.5%)	293 (69.4%)	
- ATG	389 (27.3%)	99 (9.9%)	290 (68.7%)	
- alemtuzumab	812 (57%)	809 (80.7%)	3 (0.7%)	
- missing	2	2	0	
Graft failure	1 (0.1%)	8 (1.9%)	9 (0.6%)	n.a.

*FluMel*  fludarabine/melphalan, *FluTreo* fludarabine/treosulfan, *Allo-HCT* allogeneic hematopoietic cell transplantation, *MSD* matched sibling donor, *UD* unrelated donor, *F* female, *M* male, *AML* acute myeloid leukemia, *KPS* Karnofsky performance status, *CMV* cytomegalovirus, *neg* negative, *pos* positive, *CR* complete remission, *CsA* cyclosporine A, MTX methotrexate, *MMF* mycophenolate mofetil, *TCD* T-cell depletion, *ATG* anti-thymocyte globulin, *allo-HCT* allogeneic hematopoietic cell transplantation, *GvHD* graft-versus-host disease, *NA* not assessed, *IQR* interquartile range, *CI* confidence interval, * n.a.* not assessed.

Some clinical features were remarkably different between both treatment groups. AML from patients treated with FluTreo were associated with high-risk characteristics for relapse as secondary AML (14.3% vs 23.9%, *p* < 0.0001) and adverse risk cytogenetics (10% vs 15.4%, *p* = 0.019). However, more patients in the FluTreo group were transplanted in CR1 compared with FluMel group (75.4% vs. 81.8%, *p* = 0.009). Hence, the donor distribution was different. MSD was used more frequently in FluMel (46.8% vs. 29.9%) and MUD 9/10 in FluTreo (10.6% vs. 17.3%, *p* < 0.0001). Further imbalances between the two groups are depicted in Table 1.

The two conditioning groups differed significantly regarding GvHD prophylaxis: in vivo TCD was used in 90.5% and 69.4% in FluMel and FluTreo groups, respectively. Alemtuzumab was predominantly used in FluMel (80.7% vs. 0.7%) and ATG (9.9% vs. 68.7%) was more often used in FluTreo conditioning. The frequency of use of GvHD prophylaxis treatments is shown in Table 1.

### Univariate comparison of outcomes

Graft failure was observed in 9 (0.6%) patients in the entire cohort (Table 1). Overall 673 (47.2%) patients died, 474 (47.2%) in FluMel and 199 (47.2%) in FluTreo group. NRM was 231 (23.0%) in the FluMel cohort and 113 (26.8%) in the FluTreo cohort. However, 201 (20%) and 78 (18.5%) patients died due to underlying disease in the FluMel and FluTreo cohorts, respectively. Cause of death did not differ significantly between the two groups (Suppl. Table S1).

**Fig. 1:**
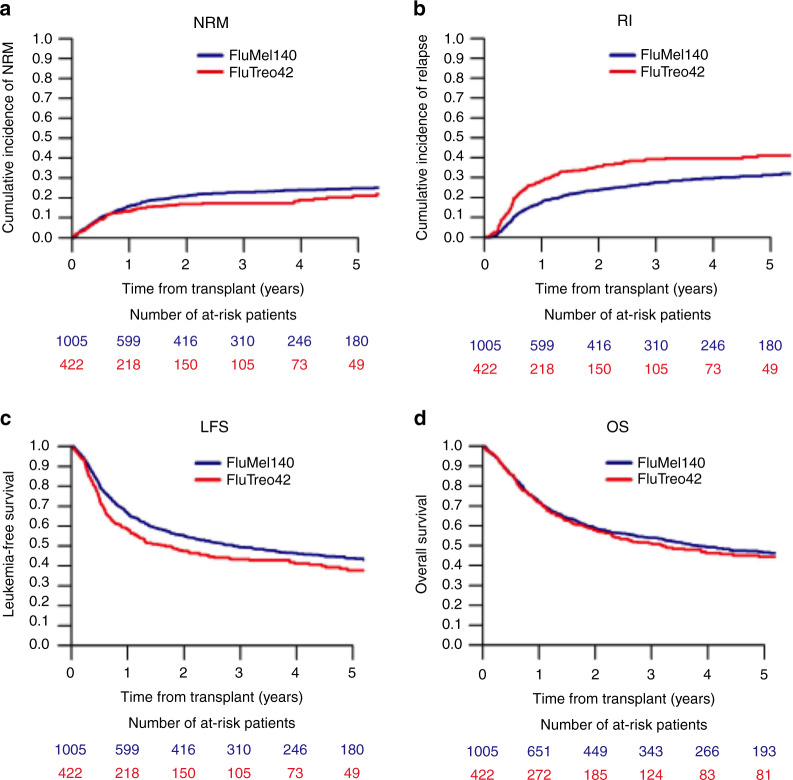
Impact of conditioning by FluMel and FluTreo on outcome. Cumulative incidences of **a** non-relapse mortality and **b** relapse by conditioning protocol are represented. Kaplan–Meier curves represent **c** leukemia-free survival and **d** overall survival by conditioning protocol. NRM non-relapse mortality; RI relapse incidence; LFS leukemia-free survival and OS overall survival; FluMel140 Fludarabine/Melphalan with a total dose 140 mg /m^2^; FluTreo42 Fludarabine/Treosulfan with a total dose of 42 g/m^2^.

In the univariate analysis of outcomes after allo-HCT at 3 years, the cumulative incidence of relapse was lower in the FluMel group compared to the FluTreo group (32.4% vs. 40.5%, *p* = 0.001). There was a corresponding lower NRM in the FluTreo group (25.7% vs. 20.2%, *p* = 0.06) but this was of borderline significance. Although the LFS was higher in FluMel than in FluTreo treated patients (49.4 % vs. 43.4%, *p* = 0.005). The OS was similar in the two treatment groups (54% vs 51.2%, *p* = 0.49) (Table 2A, B, Fig. 1).

**Table 2:** Univariate analysis of conditioning regimen and (A) outcome variables and (B) acute and chronic GvHD incidence.

A. Outcome variables					
	**3 years**				
	**Relapse**	**NRM**	**LFS**	**OS**	**GRFS**
FluMel	32.4%[29.2–35.7]	25.7%[22.8–28.6]	49.4%[46–52.7]	54%[50.6–57.2]	44.4%[41–47.7]
FluTreo	40.5%[35.4–45.6]	20.2%[16.2–24.5]	43.4%[38.3–48.5]	51.2%[45.8–56.3]	31.9%[27.1–36.9]
*P* value	0.001	0.06	0.005	0.49	0.001

B. Acute and chronic GvHD incidence				
	**180 days**		**3 years**	
	**Acute GVHD II-IV**	**Acute GVHD III-IV**	**chronic GVHD**	**ext. chronic GVHD**
FluMel	24.8%[22.1–27.6]	8.3%[6.6–10.1]	31.8%[28.7–34.9]	10.4%[8.5–12.6]
FluTreo	19.7%[15.9–23.7]	10%[7.3–13.1]	35.4%[30.5–40.3]	20.2%[16.1–24.6]
*P* value	0.037	0.34	0.16	0.001

Univariate analysis of outcome variables by conditioning.

*FluMel* fludarabine/melphalan, *FluTreo* fludarabine/treosulfan, *GvHD* Graft-versus-host disease, *ext.* extensive, *NRM* non-relapse mortality, *LFS* leukemia-free survival, *OS* overall survival, *GRFS* GvHD-free relapse-free survival.

### Multivariate comparison of outcomes

In the multivariate analysis with the presence of variables known to influence outcome in the univariate analysis (Suppl. Table S2) as older age, adverse cytogenetics, CR2 + remission status, MUD, female donor to male recipient and Karnofsky performance score (KPS), FluTreo was associated with higher risk of relapse (HR 1.46, 95% CI 1.15–1.85, *p* = 0.002) and lower risk of NRM (HR, 0.66 95% CI 0.47–0.93, *p* = 0.018) as compared to FluMel. However, there were no significant differences in LFS ((HR 1.1, 95% CI 0.91–1.33, *p* = 0.31), GFRS (HR 1.14, 95% CI 0.93–1.4, *p* = 0.21) or OS (HR 0.9, 95% CI 0.73–1.11, *p* = 0.31) between treatment groups (Table 3).

**Table 3:** Multivariate analysis of outcome variables.

	Relapse		NRM		LFS		OS		GRFS	
	HR (95% CI)	*p* value	HR (95% CI)	*p* value	HR (95% CI)	*p* value	HR (95% CI)	*p* value	HR (95% CI)	*p* value
FluTreo vs FluMel	1.46 (1.15–1.85)	0.002	0.66 (0.47–0.93)	0.018	1.1 (0.91–1.33)	0.31	0.9 (0.73–1.11)	0.31	1.14 (0.93–1.4)	0.21
Age (per 10 years)	1 (0.9–1.11)	0.99	1.57 (1.34–1.83)	<0.0001	1.16 (1.06–1.27)	0.001	1.21 (1.1–1.33)	<0.0001	1.1 (1.01–1.19)	0.029
Year of allo-HCT	0.96 (0.92–0.99)	0.012	0.98 (0.94–1.03)	0.51	0.97 (0.95–1)	0.078	0.98 (0.95–1.01)	0.27	1 (0.97–1.03)	0.98
secAML	0.94 (0.72–1.24)	0.68	1.14 (0.84–1.56)	0.4	1.04 (0.84–1.29)	0.69	1.12 (0.9–1.4)	0.32	1.06 (0.87–1.3)	0.55
adverse cytogenetics	2.8 (2.18–3.6)	<0.0001	1.46 (0.97–2.19)	0.068	2.2 (1.77–2.74)	0.0001	2.08 (1.65–2.64)	<0.0001	2 (1.62–2.47)	<0.0001
CR2 + vs CR	1.26 (0.98–1.6)	0.067	1.4 (1.06–1.84)	0.017	1.34 (1.11–1.62)	0.002	1.32 (1.08–1.61)	0.006	1.24 (1.04–1.49)	0.018
Donor reference =MSD	1		1		1		1		1	
MUD 10/10	0.98 (0.77–1.24)	0.85	2.03 (1.49–2.76)	<0.0001	1.37 (1.13–1.66)	0.002	1.45 (1.18–1.79)	0.0004	1.23 (1.02–1.49)	0.028
MUD 9/10	1.12 (0.78–1.59)	0.54	4.63 (3.17–6.76)	<0.0001	2.2 (1.7–2.85)	<0.0001	2.62 (2–3.43)	<0.0001	2.21 (1.71–2.84)	<0.0001
Female to male	0.95 (0.71–1.27)	0.74	1.88 (1.4–2.53)	<0.0001	1.36 (1.1–1.68)	0.004	1.39 (1.11–1.75)	0.004	1.29 (1.05–1.58)	0.014
KPS ≥ 90	0.92 (0.72–1.19)	0.54	0.7 (0.52–0.93)	0.015	0.77 (0.63–0.93)	0.006	0.72 (0.59–0.88)	0.001	0.85 (0.7–1.03)	0.094
Pat. CMV pos	1.11 (0.88–1.4)	0.37	1.22 (0.91–1.63)	0.18	1.16 (0.96–1.4)	0.12	1.11 (0.91–1.36)	0.31	1.11 (0.92–1.32)	0.27
Don. CMV pos	0.98 (0.79–1.22)	0.87	0.99 (0.76–1.29)	0.93	1.03 (0.86–1.22)	0.78	1 (0.83–1.21)	0.98	0.97 (0.82–1.14)	0.72
in vivo TCD	1.07 (0.79–1.46)	0.66	0.63 (0.43–0.92)	0.017	0.85 (0.67–1.09)	0.21	0.83 (0.63–1.08)	0.16	0.66 (0.52–0.83)	0.0005
Frailty		0.65		0.085		0.88		0.26		0.14

Multivariate analysis of outcome variables depending on conditioning.

*FluMel*  fludarabine/melphalan, *FluTreo* fludarabine/treosulfan, *GvHD* graft-versus-host disease, *NRM* non-relapse mortality, *LFS* leukemia-free survival, *OS* overall survival, *GRFS* GvHD-free relapse-free survival, *secAML* secondary acute myeloid leukemia, *CR* complete remission, *MSD* matched sibling donor, *MUD* matched unrelated donor, *KPS* Karnofsky performance status, *Pat.* patient, *Don.* donor, *CMV* cytomegalovirus, *TCD T* cell depletion, *HR* hazard ratio, *CI* confidence interval.

### Subgroup analysis by GvHD prophylaxis

To assess the impact of the GvHD prophylaxis on outcome, we performed a subgroup analysis of patients without any in vivo TCD (FluMel 95 patients, FluTreo 129 patients) and with ATG (FluMel 99 patients, FluTreo 290 patients) comparing both conditioning protocols. As only 3 patients of the FluTreo group received alemtuzumab, it was not possible to adjust the comparison on this variable. Univariate analysis stratified on in vivo TCD with ATG or no in vivo TCD were consistent with the entire population (Suppl. Table S3).

### Subgroup analysis by age

To identify a patient population, which might benefit most from a specific conditioning regimen, we performed a subgroup analysis of patients aged <55 years and aged ≥55 years. Altogether, 527 patients aged <55 years (379 patients with FluMel and 148 patients with FluTreo) and 900 patients aged ≥55 years (626 patients with FluMel and 274 patients with FluTreo) were included. Similar to the previously described results in the entire cohort, no differences were found by conditioning in both patient age subgroups regarding outcome and GvHD incidence, suggesting that both protocols are also suitable for older patients (Suppl. Tables S4, S5).

### Matched pair case analysis

In order to minimize the effect of confounding factors we performed a matched case analysis. For each patient receiving FluTreo, we were able to identified either one (*n* = 144) or two (*n* = 219) controls who received FluMel using exact and propensity-score matching. We included in this analysis a total of 582 patients in FluMel and 363 patients in FluTreo groups. Exact matching was performed for status at allo-HCT (CR1/CR2), cytogenetics (adverse vs other) type of AML (de novo or secondary), donor type (MSD, UD 10/10 or UD + 9/10). The propensity score was based on patient age and in vivo TCD. The patients were well matched, with standardized mean difference estimates of less than 5% for all matched parameters except age. To take into account for similarity within each pair, a cluster was used and comparison was adjusted on patient age. The results are consistent with those found in the entire population, but GRFS was significantly lower in the FluTreo group (Table 4, Suppl. Fig. S1).

**Table 4:** Matched pair case analysis.

	FluMel (*n* = 582)	FluTreo (*n* = 363)	HR (95% CI)	*p* value
Relapse	30.5% [26.3–34.9]	40.6% [35.1–46]	1.51 (1.21–1.89)	0.0003
NRM	31.9% [27.8–36.2]	19.7% [15.4–24.4]	0.69 (0.52–0.92)	0.01
LFS	44% [39.5–48.5]	43.6% [38–49]	1.12 (0.94–1.34)	0.21
OS	48.3% [43.7–52.7]	51.7% [45.9–57.1]	0.92 (0.76–1.12)	0.43
GRFS	39.5% [35–43.9]	32.2% [27–37.5]	1.25 (1.07–1.47)	0.005
Acute GVHD II-IV	30.4% [26.6–34.3]	19.3%[15.3–23.6]	0.59 (0.44–0.79)	0.0004
Acute GVHD III-IV	10.6% [8.2–13.3]	9.5%[6.7–12.9]	0.88 (0.58–1.34)	0.56
Chronic GVHD	34.3% [30–38.5]	34%[28.8–39.2]	1.09 (0.86–1.38)	0.49
Ext. chronic GVHD	11.7% [9–14.8]	19.6%[15.3–24.4]	1.69 (1.17–2.44)	0.005

*FluMel*  fludarabine/melphalan, *FluTreo* fludarabine/treosulfan, *NRM* non-relapse mortality, *LFS* leukemia-free survival, *OS* overall survival, *GRFS* GvHD-free relapse-free survival, *GvHD* graft-versus-host disease, *HR* hazard ratio, *CI* confidence interval, *ext* extensive.

## Discussion

Due to the increasing age of patients at allo-HCT and frequently, presence of comorbidities, a pressing research question is to identify the right conditioning protocol based on individual patient characteristics.

Whereas previously, efforts have focused on decreasing conditioning intensity as much as possible, recent analyses have pointed towards the value of adequate dosing intensity. Randomized clinical trials and registry studies have shown that by selecting AML patients by age and hematopoietic cell transplantation–comorbidity index (HCT-CI) score receiving MAC, outcomes improved compared to a similar patient population receiving RIC [14, 15]. Hence, AML patients in CR with minimal residual disease (MRD) detectable by next-generation sequencing technology have also improved outcome with MAC compared with RIC [16]. However, the conditioning intensity has been reduced while preserving outcomes in other hematological malignancies such as myelodysplastic syndrome (MDS) [17] as well as the TBI dose in AML patients [18, 19].

Conditioning intensity is a crucial factor, influencing the prognosis of patients in the allo-HCT setting. Recently, FluTreo (total dose 30 g/m^2^) was compared with FluBu2 in a prospective randomized clinical trial for patients with AML/MDS and improved outcome was observed in patients treated with FluTreo [20]. In another prospective trial, patients with an HCT-CI ≤ 4 were randomized to receive MAC including FluBu4 (fludarabine 120–180 mg/m^2^, busulfan 16 mg/kg orally or 12.8 mg/kg intravenously, 4 days), Busulfan/Cyclophosphamide (Bu4Cy, busulfan 16 mg/kg orally or 12.8 mg/kg intravenously, 4 days, and cyclophosphamide 120 mg/kg), total body irradiation/Cyclophosphamide (TBI/Cy, 12–14.2 Gy and cyclophosphamide 120 mg/kg) or RIC including FluBu2 (fludarabine 120–180 mg/m^2^, busulfan 8 mg/kg orally or 6.4 mg/kg intravenously, 2 days) and FluMel (fludarabine 120–180 mg/m^2^, melphalan total dose ≤150 mg/m^2^). Patients receiving MAC showed significantly better outcomes than patients receiving RIC, although these data may have been confounded by the heterogenous conditioning regimes used in each group and the smaller proportion (20%) of patients conditioned with FluMel in the RIC group [14]. In the FIGARO trial, it was demonstrated that FLAMSA-Bu (Fludarabine, Cytarabine 2 g/m^2^ × 4 days, AMSA 100 mg/m^2^ × 4 days, Busulfan -total dose 11.2 mg/kg) did not improved outcome of patients conditioned with FluBu2 neither in MRD positive nor negative patients with AML [21]. However, it has been discussed that that FluBu2, FluTreo, FLAMSA-Bu, and Flu/Mel are equivalent and it has been proposed, that efforts to improve transplant outcome should rather be focused on the important strategic point of pre- and post- allo-HCT modifications [2]. Here, we focused on two well-established conditioning protocols (with an intermediate TCI score), which aimed to reduce toxicity [5] based on fludarabine combined with either melphalan or treosulfan. We analyzed the most commonly used doses in the EBMT registry data, namely fludarabine at 150 mg/m^2^ and melphalan at 140 mg/m^2^ (FluMel), and fludarabine at 150 mg/m^2^ and treosulfan at 42 g/m^2^ (FluTreo).

In the multivariate analysis as well as in matched case analysis, patients treated with FluTreo showed a higher incidence of relapse but lower NRM compared to patients treated with FluMel, suggesting a better antileukemic effect but higher toxicity with FluMel. There was no significant difference in terms of LFS and OS. Recent retrospective analysis showed that FluMel has a similar anti-leukemic activity compared with other more intense protocols such as FluBu4 and Bu4Cy. FluMel demonstrated better anti-leukemic activity but also higher toxicity compared to other less intense protocols including FluBu2 in registry-based studies [7, 8]. With respect to the subgroup analyses in patients aged <55 years and ≥55 years, there were no significant differences between FluMel and FluTreo regarding outcome variables and GvHD incidence, demonstrating that both protocols are also suitable for older patients (Suppl. Tables S4, S5). This might also be influenced by selection bias, which patients were fit for more or less intense protocols.

Several factors are known to contribute to outcome independently of the conditioning regimen used such as age, KPS score, remission status (CR1 vs CR2+), adverse cytogenetic risk, unrelated donor, female donor to male recipient, in vivo TCD and pre-transplant MRD. In vivo TCD (alemtuzumab or ATG) was more often used in the FluMel group than in the FluTreo group. In the former, in vivo TCD was given not only to patients receiving a transplant from an unrelated donor but also from a related donor. Previously, unrelated, and related donor recipients receiving ATG have shown better outcomes in terms of GRFS compared to standard GvHD prophylaxis [22–24].

This study has several other limitations. This is an analysis of a retrospective registry-based study. Although the gold standard method of comparing conditioning protocols on outcomes after allo-HCT is the randomized controlled trial, the performance of such a clinical trial with enough patients to elucidate the appropriate conditioning protocol for a patient populations in an allo-HCT setting is no trivial matter. The value of a registry database analysis is the large number of patients. The conditioning protocols are country/center dependent. FluMel, mainly in combination with alemtuzumab for in vivo TCD as GvHD prophylaxis, and is most commonly used in United Kingdom, Belgium, and Switzerland. However, FluTreo in combination with ATG for in vivo TCD as GvHD prophylaxis, is more frequently used in Germany, Finland, Sweden, and Italy. As mentioned above, distribution of donor type was also imbalanced. Allo-HCT from an MSD was more frequently performed with FluMel (46.8%) conditioning than with FluTreo (29.9%). GvHD prophylaxis based on in vivo TCD (alemtuzumab or ATG) was more often used together with FluMel (90.5%) than with FluTreo (69.4%), which might also reflect center/country differences in the combination of conditioning with a specific GvHD prophylaxis regimen. The source of ATG (Grafalon versus Thymoglobulin) was not always documented in the ALWP registry, which might also have affected the GvHD incidence, and therefore, outcome. We included in vivo TCD in the multivariate analysis, which was associated with decreased NRM and GFRS. However, we did not differentiated between patients receiving alemtuzumab (more often used in FluMel patients with 80.7%) und ATG (more often used in FluTreo patients with 68.7%) among patients receiving in vivo TCD in the entire cohort. However, in a subgroup analysis, univariate subgroup analysis stratified on in vivo TCD with ATG or no in vivo TCD were consistent with the entire population (Suppl. Table S3). Only 3 patients in the FluTreo group received alemtuzumab, so it was not possible to adjust the comparison on this variable in statistical analysis. Another limitation of the study is the missing data in cytogenetics of included patients and the absence of MRD and next-generation sequencing data, which is pivotal in determining transplant outcome. We acknowledge, that we focus in this work only on conditioning protocols and that interventions prior allo-HCT as chemotherapy before conditioning [21] and after allo-HCT as pre-emptive donor lymphocyte infusions [25], hypomethylating agents [26], and MRD-driven therapeutic approaches [27] are playing an increasing role in the management of patients undergoing allo-HCT. Altogether, these data should therefore be interpreted with caution.

In this study, at 3 years after allo-HCT, we show an NRM of 25% with FluMel and 20% with FluTreo and both protocols have resulted in an anti-leukemic effect with relapse rates of ~32% with FluMel and 40% with FluTreo, results similar to previous studies [6, 7, 20]. More effort should be made to increase the intensification of the protocols without affecting toxicity. Our data may serve as a basis for further comparisons, e.g., with regimens containing additional alkylating/cytostatic agents.

In summary, we have characterized and compared two of the most frequently used conditioning protocols with an intermediate TCI score, based on a combination of a single alkylating agent with fludarabine (FluMel and FluTreo), in patients with AML in CR, using registry data from the ALWP/EBMT. Both protocols showed similar outcomes regarding OS.

## Supplementary information

Supplementary tables

Supplementary Figure 1

## Data Availability

The datasets generated during and/or analyzed during the current study are available upon reasonable request from the corresponding authors.
