# Supplementary material for: Comparison of fludarabine–melphalan and fludarabine–treosulfan as conditioning prior to allogeneic hematopoietic cell transplantation—a registry study on behalf of the EBMT Acute Leukemia Working Party
Source: Bone Marrow Transplant. 2022 May 14;57(8):1269–76. doi: 10.1038/s41409-022-01646-1 (PMC9352579; doi:10.1038/s41409-022-01646-1)
Supplement: Supplementary file 1 — Supplementary tables [file 41409_2022_1646_MOESM1_ESM.pdf]

Supplementary Tables

Supplementary Table 1. Cause of death

Variable	Entire cohort	FluMel	FluTreo
**N**	1427	1005	422
Relapse, n(%)	279 (19.5)	201 (20.0)	78 (18.5)
NRM, n(%)	344 (24.1)	231 (23.0)	113 (26.8)
Missing, n(%)	50 (3.5)	42 (4.2)	8 (1.9)
Total Deaths, n (%)	673 (47.2%)	474 (47.2)	199 (47.2)

Suppl. Table 1. Cause of death according to conditioning regimen.

Table shows the number and percentage of a specific cause of death from total deaths in each cohort. Non-Relapse Mortality (NRM) includes infections, GvHD, secondary malignancies, interstial pneumonitis and central nervous toxicity as the most frequent cause of death. FluMel, fludarabine/melphalan; FluTreo, fludarabine/treosulfan; VOD, veno-occlusive disease; GvHD, graft-versus-host disease; MOF, multi-organ failure; CNS, central nervous system; allo-HCT, allogeneic hematopoietic cell transplantation.

Suppl. Table 2. Univariate analysis of patient and transplant characteristics

		3 years				
		**Relapse**	**NRM**	**LFS**	**OS**	**GRFS**
**Patient age**	**age<median**	34%[30.2-37.9]	17.1%[14.3-20.2]	54.7%[50.7-58.5]	60.3%[56.3-64.1]	47%[43-50.9]
	**age>median**	35.8%[31.8-39.7]	31.1%[27.4-34.9]	40.4%[36.4-44.3]	45.9%[41.8-49.8]	34.4%[30.6-38.3]
	**P value**	0.75	**0.001**	**0.001**	**0.001**	**0.001**
**Age >=<55y**	**age<55y**	35.6%[31.1-40]	15.6%[12.5-19.1]	54.1%[49.5-58.5]	59.4%[54.8-63.8]	47.5%[42.9-52]
	**age>=55y**	34.5%[31.1-38]	29.2%[25.9-32.5]	43.6%[40.1-47.2]	49.4%[45.8-52.9]	36.7%[33.2-40.1]
	**P value**	0.54	**0.001**	**0.001**	**0.001**	**0.001**
**Year of allo-HCT**	**Year<median**	37.1%[33.1-41.1]	25.4%[21.9-29]	45.9%[41.8-50]	52.2%[48.1-56.3]	41%[36.9-45.1]
	**Year>median**	32.1%[28.4-35.8]	22.4%[19.3-25.7]	49.3%[45.4-53.1]	53.8%[49.8-57.6]	40.9%[37.2-44.7]
	**P value**	0.18	0.41	0.35	0.53	0.42
**Status at transplant**	**CR1**	34.8%[31.7-38]	22%[19.4-24.8]	49.8%[46.6-53]	55.2%[52-58.4]	42.3%[39.1-45.5]
	**CR2+**	34.9%[29.3-40.5]	30.9%[25.7-36.3]	40.3%[34.6-45.9]	46.4%[40.5-52]	35.4%[29.9-41]
	**P value**	0.88	**0.001**	**0.001**	**0.001**	**0.031**
**AML type**	**de novo**	35.4%[32.4-38.5]	23.7%[21.1-26.4]	47.7%[44.6-50.8]	53.4%[50.2-56.4]	41%[37.9-44]
	**secAML**	32.3%[26-38.7]	25.9%[20.3-31.9]	47.1%[40.3-53.7]	52%[45.1-58.4]	39.7%[33.1-46.2]
	**P value**	0.41	0.35	0.73	0.38	0.46
**Cytogene-tics**	**favourable**	32.4%[20.3-45.1]	21.5%[11.7-33.2]	46.1%[32.4-58.7]	54%[39.6-66.3]	39.6%[26.6-52.4]
	**Intermed.**	30.1%[26.2-34.2]	24.7%[21-28.6]	51.5%[47.2-55.6]	56.2%[51.9-60.3]	43.1%[38.9-47.2]
	**adverse**	61.1%[52-69]	20.4%[14.2-27.3]	25.1%[18-32.8]	34.1%[26.2-42.2]	18.1%[12.1-25.1]
	**NA/failed**	32.3%[28.1-36.5]	24.7%[21-28.6]	50.3%[45.8-54.6]	55.4%[50.9-59.7]	45.5%[41-50]
	**P value**	**0.001**	0.95	**0.001**	**0.001**	**0.001**
	**not adverse**	31.6%[28.7-34.4]	24.5%[22-27.1]	50.5%[47.5-53.4]	55.6%[52.6-58.6]	43.7%[40.7-46.7]
	**adverse**	61.1%[52-69]	20.4%[14.2-27.3]	25.1%[18-32.8]	34.1%[26.2-42.2]	18.1%[12.1-25.1]
	**P value**	**0.001**	0.62	**0.001**	**0.001**	**0.001**
**Donor**	**MSD**	36.6%[32.3-40.9]	17%[13.8-20.4]	55.2%[50.8-59.4]	61.6%[57.3-65.7]	46.4%[42-50.7]
	**MUD 10/10**	33.8%[29.8-37.8]	27.2%[23.5-31]	44.3%[40.1-48.4]	49.8%[45.5-53.9]	39.7%[35.6-43.8]
	**MUD 9/10**	33.2%[25.7-40.8]	35.8%[28.6-43.1]	34.7%[27.3-42.2]	37.7%[30.1-45.4]	25.9%[19.2-33.1]
	**P value**	0.58	**0.001**	**0.001**	**0.001**	**0.001**
**Karnofsky score**	**<90**	31.6%[25.7-37.6]	30.5%[24.6-36.5]	41.1%[34.7-47.3]	45.4%[39-51.7]	37.2%[31-43.4]
	**>=90**	35.7%[32.6-38.9]	22%[19.4-24.7]	49.4%[46.2-52.6]	55.6%[52.3-58.7]	42.1%[38.9-45.3]
	**P value**	0.73	**0.002**	**0.003**	**0.001**	0.08
**Patient sex**	**male**	37.2%[33.4-41]	26%[22.6-29.5]	42.9%[39-46.7]	48.7%[44.7-52.6]	37.5%[33.7-41.3]
	**female**	32.3%[28.4-36.3]	21.9%[18.6-25.4]	52.8%[48.7-56.8]	58.1%[54-62]	44.3%[40.2-48.3]
	**P value**	0.07	0.17	**0.007**	**0.016**	0.1
**Donor sex**	**male**	36.5%[33.1-40]	22.9%[20.1-25.9]	46.6%[43.1-50]	51.7%[48.2-55.1]	40.4%[36.9-43.8]
	**female**	32.2%[27.6-36.8]	25.8%[21.6-30.2]	49.1%[44.2-53.9]	55.7%[50.8-60.4]	41.4%[36.5-46.1]
	**P value**	0.13	0.29	0.52	0.38	0.8
**Female to male combinatio**	**No**	35.6%[32.6-38.7]	22.7%[20.2-25.4]	48.2%[45.1-51.3]	53.5%[50.4-56.6]	41.2%[38.1-44.2]

**n**						
	**Yes**	31.4%[24.9-38.1]	30.5%[24.3-36.9]	44.4%[37.4-51.2]	51.3%[44.2-58]	38.3%[31.5-45.1]
	**P value**	0.18	**0.008**	0.31	0.34	0.49
**Patient CMV**	**CMV neg.**	31.9%[27.4-36.5]	23.4%[19.4-27.7]	52.5%[47.5-57.1]	55.6%[50.7-60.3]	45.9%[41.1-50.7]
	**CMV pos**	36.2%[32.8-39.7]	24.4%[21.5-27.4]	45.3%[41.8-48.7]	52%[48.5-55.4]	38.2%[34.8-41.6]
	**P value**	0.12	0.34	**0.009**	0.14	**0.019**
**Donor CMV**	**CMV neg.**	35%[31-39]	22.7%[19.4-26.2]	50.4%[46.3-54.4]	54.4%[50.3-58.4]	43.2%[39.1-47.1]
	**CMV pos**	34.8%[31-38.7]	24.9%[21.5-28.4]	45.2%[41.2-49.1]	52.5%[48.5-56.4]	38.7%[34.8-42.6]
	**P value**	0.59	0.29	**0.049**	0.25	0.13
***In vivo* TCD**	**No**	38.3%[30.9-45.7]	24.7%[18.5-31.4]	45.8%[38.2-53]	54.2%[46.5-61.2]	32%[25.1-39.1]
	**Yes**	34.3%[31.3-37.2]	24%[21.4-26.6]	48%[44.9-51]	53%[49.9-56]	42.2%[39.2-45.2]
	**P value**	0.25	0.84	0.37	0.95	**0.001**

Suppl. Table 2 Univariate analysis of patient and transplant characteristics. NRM, non-relapse mortality; LFS, leukemia-free survival; OS, overall survival; GRFS, GvHD-free, relapse-free survival; GvHD, graft-versus-host disease, ext, extensive; sec. AML, secondary acute myeloid leukemia; intermed., intermediate; CR, complete remission; MSD, matched sibling donor; MUD, matched unrelated donor; KPS, Karnofsky performance status; Pat, patient; Don, donor; CMV, cytomegalovirus; TCD, T-cell depletion; allo-HCT, allogeneic hematopoietic cell transplantation; NA, not assessed; M, male; F, female.

Suppl. Table 3. Univariate subgroup analysis (GvHD prophylaxis)

		3 years				
		**Relapse**	**NRM**	**LFS**	**OS**	**GRFS**
**no TCD**	**FluMel (n=95)**	31.9%[20.5-43.9]	35%[24.1-46.2]	44.1%[32.2-55.3]	48.7%[36.8-59.5]	37.6%[26.2-48.9]
	**FluTreo (n=129)**	43.5%[33.7-53]	17.2%[10.5-25.3]	46.8%[37-56.1]	58.2%[48-67]	28.2%[19.8-37.3]
	**p value**	**0.031**	**0.003**	0.82	0.12	0.43
**ATG**	**FluMel (n=99)**	19.1%[11.7-28]	31.5%[21.9-41.4]	49.4%[38.5-59.4]	51%[39.9-61.1]	42.9%[32.2-53]
	**FluTreo (n=290)**	39.2%[33.2-45.1]	20.7%[16-25.9]	42.6%[36.5-48.6]	48.9%[42.5-55]	33.7%[27.9-39.7]
	**p value**	**0.002**	**0.02**	0.58	0.66	0.47

Suppl. Table 3. Univariate subgroup analysis (GvHD prophylaxis). FluMel, fludarabine/melphalan; FluTreo, fludarabine/treosulfan; NRM, non-relapse mortality; LFS, leukemia-free survival; OS, overall survival; GRFS, GvHD-free, relapse-free survival; TCD, T cell depletion; ATG, anti-thymocyte globulin;

Suppl. Table 4. Univariate subgroup analysis (age <55 years)

	3 years				
	Relapse	NRM	LFS	OS	GRFS
**FluMel (n=379)**	32.8%[27.7-37.9]	17%[13.2-21.3]	56.8%[51.3-61.9]	59.7%[54.1-64.8]	52.8%[47.2-58]
**FluTreo (n=148)**	42.8%[33.9-51.4]	11.6%[6.9-17.6]	46.9%[37.9-55.3]	58.9%[49.7-67]	33.7%[25.6-42.1]
**P value**	**0.005**	0.24	**0.015**	0.8	**0.001**

Suppl. Table 4. Univariate subgroup analysis (age<55 years). FluMel, fludarabine/melphalan; FluTreo, fludarabine/treosulfan; GvHD, graft-versus-host disease; NRM, non-relapse mortality; LFS, leukemia-free survival; OS, overall survival; GRFS, GvHD-free, relapse-free survival.

Suppl. Table 5. Univariate subgroup analysis (age ≥55 years)

	3 years				
	**Relapse**	**NRM**	**LFS**	**OS**	**GRFS**
**FluMel (n=626)**	32.4%[28.3-36.6]	31.1%[27.2-35.2]	44.6%[40.2-48.8]	50.4%[46.1-54.6]	39.1%[34.9-43.3]
**FluTreo (n=274)**	39.4%[33.1-45.6]	24.6%[19.2-30.4]	41.5%[35.1-47.7]	47%[40.3-53.3]	30.9%[25-37]
**P value**	**0.005**	0.08	0.12	0.61	**0.011**

Suppl. Table 5. Univariate subgroup analysis (age≥55 years).

FluMel, fludarabine/melphalan; FluTreo, fludarabine/treosulfan; GvHD, graft-versus-host disease; NRM, non-relapse mortality; LFS, leukemia-free survival; OS, overall survival; GRFS, GvHD-free, relapse-free survival.
