# Supplementary material for: Comparison of fludarabine–melphalan and fludarabine–treosulfan as conditioning prior to allogeneic hematopoietic cell transplantation—a registry study on behalf of the EBMT Acute Leukemia Working Party
Source: Bone Marrow Transplant. 2022 May 14;57(8):1269–76. doi: 10.1038/s41409-022-01646-1 (PMC9352579; doi:10.1038/s41409-022-01646-1)
Supplement: Supplementary file 2 — Supplementary Figure 1 [file 41409_2022_1646_MOESM2_ESM.pdf]

Supplementary Figure 1

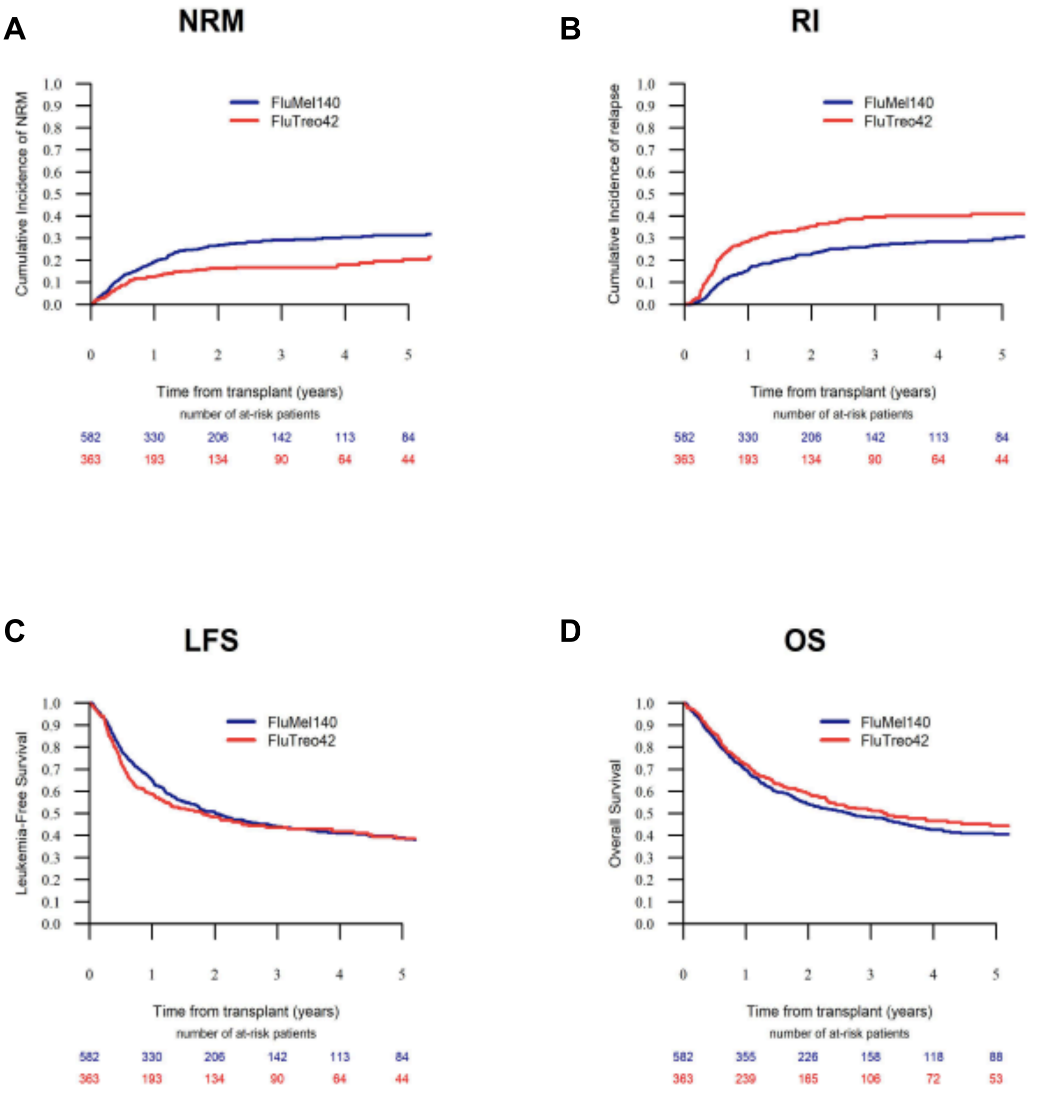

Supplementary Figure 1. Matched case analysis. Cumulative incidences of **(A)** non-relapse mortality and **(B)** relapse by conditioning protocol are represented. Kaplan–Meier curves represent **(C)** leukemia-free survival and **(D)** overall survival by conditioning protocol. NRM, non-relapse mortality; RI, relapse incidence; LFS, leukemia-free survival and OS, overall survival; FluMel140, Fludarabine/Melphalan with a total dose 140 mg /m2; FluTreo42, Fludarabine/Treosulfan with a total dose of 42 g/m2.
